# Promising Biomarkers in Head and Neck Cancer: The Most Clinically Important miRNAs

**DOI:** 10.3390/ijms23158257

**Published:** 2022-07-26

**Authors:** Arsinoe C. Thomaidou, Panagiota Batsaki, Maria Adamaki, Maria Goulielmaki, Constantin N. Baxevanis, Vassilis Zoumpourlis, Sotirios P. Fortis

**Affiliations:** 1 Biomedical Applications Unit, Institute of Chemical Biology, National Hellenic Research Foundation (NHRF), 11635 Athens, Greece; arsithomaidu@gmail.com (A.C.T.); madamaki@eie.gr (M.A.); 2 Cancer Immunology and Immunotherapy Center, Saint Savas Cancer Hospital, 11522 Athens, Greece; pmpatsaki@agsavvas-hosp.gr (P.B.); mgoulielmaki@eie.gr (M.G.); baxevanis@ciic.gr (C.N.B.)

**Keywords:** miRNAs, HNC, biomarkers, signatures, HPV, clinical trials

## Abstract

Head and neck cancers (HNCs) comprise a heterogeneous group of tumors that extend from the oral cavity to the upper gastrointestinal tract. The principal etiologic factors for oral tumors include tobacco smoking and alcohol consumption, while human papillomavirus (HPV) infections have been accused of a high incidence of pharyngeal tumors. Accordingly, HPV detection has been extensively used to categorize carcinomas of the head and neck. The diverse nature of HNC highlights the necessity for novel, sensitive, and precise biomarkers for the prompt diagnosis of the disease, its successful monitoring, and the timely prognosis of patient clinical outcomes. In this context, the identification of certain microRNAs (miRNAs) and/or the detection of alterations in their expression patterns, in a variety of somatic fluids and tissues, could serve as valuable biomarkers for precision oncology. In the present review, we summarize some of the most frequently studied miRNAs (including miR-21, -375, -99, -34a, -200, -31, -125a/b, -196a/b, -9, -181a, -155, -146a, -23a, -16, -29, and let-7), their role as biomarkers, and their implication in HNC pathogenesis. Moreover, we designate the potential of given miRNAs and miRNA signatures as novel diagnostic and prognostic tools for successful patient stratification. Finally, we discuss the currently ongoing clinical trials that aim to identify the diagnostic, prognostic, or therapeutic utility of miRNAs in HNC.

## 1. Introduction

Head and neck squamous cell carcinoma (HNSCC) is one of the six most common cancers worldwide, counting more than 850,000 new cases and 400,000 deaths, annually [1]. It is considered as one of the most aggressive cancer types, with serious repercussions for patient quality of life, mainly due to advanced local disease and low responsiveness to treatment [2]. HNSCC is an epithelial malignancy that comprises a highly heterogeneous group of tumors located in the oral cavity, larynx, nasopharynx, oropharynx, and hypopharynx [3]. The most common HNSCC risk factors are related to tobacco smoking and alcohol consumption [1]. Human papillomavirus (HPV) infection is also considered a major causative agent, especially in tumors located in the oropharynx [1]. Popular treatment regimens involve surgical removal, radiotherapy (RT), and chemotherapy (CT). Despite recent advances, such as new targeted therapies that include immune checkpoint inhibitors [3], existing therapeutic modalities are often unsuccessful, usually accompanied by a high post-operative recurrence rate and no obvious improvement in five-year survival rates [1,4]. Therefore, HNSCC patients still witness poor prognosis and survival rates remain low, since up to 25% of the affected individuals develop local recurrence or distant metastases [5], as well as lymph node metastases (LNM) [6].

Abnormal expression and accumulation of mutations in oncogenes or tumor suppressor genes are the major steps for head and neck cancer (HNC) development and tumor progression [7,8]. HNC cells have the ability to communicate with external microenvironments and to escape detection by the host immune system. Considering the complexity of HNSCC and the urgent need for improved diagnosis and prognosis of the disease, most researchers have focused on finding novel, clinically relevant biomarkers. Previous studies have shown that certain microRNAs (miRNAs) in HNSCC are differentially expressed, functioning as regulators of tumor suppressors or oncogenes [9,10].

MiRNAs make up a group of non-protein-coding RNAs approximately 18–22 nucleotides long [11]. The biogenesis of miRNAs arises in the nucleus, where they are subsequently exported from into the cytoplasm for further processing [12]. An abundance of human miRNAs has been reported to date, with the majority being able to target and regulate multiple genes. Briefly, miRNAs bind to the 3′-UTR of mRNAs, thereby inducing cleavage and post-transcriptional silencing of these target molecules [13,14]. MiRNAs can control diverse biological functions including embryogenesis, cellular development, and homeostasis, but also appear to play a role in tumorigenesis and cancer development, functioning as regulators of either oncogenes or tumor suppressor genes [15,16]. Aberrantly expressed miRNAs are implicated in several oncogenic processes, including cell proliferation, differentiation, migration, and apoptosis [17,18]. Recently, an increasing number of studies have focused on the differential expression of certain miRNAs in various solid tumors, such as breast [19], lung [20], prostate [21], colon [22], ovarian [23], and HNC [9]. Deregulated miRNA expression levels can be tissue-specific and miRNAs can be characterized as oncogenic (OncomiRs), or tumor suppressor miRNAs, depending on the genes that they regulate and their mode of action. However, it is important to note that each miRNA could exhibit a different expression pattern and function in different cancer types. Furthermore, it has been shown that miRNA signatures can serve as biomarkers for diagnosis and prognosis, and for predicting a patient’s response to treatment [24,25]. There is also enough evidence to suggest that a significant number of miRNAs are abnormally expressed during HNSCC tumorigenesis and progression, either functioning as OncomiRs or as tumor suppressors [26].

In the present review, we summarize some of the most extensively studied miRNAs, the expression levels of which are aberrantly altered and, in this way, involved in the pathogenesis of HNSCC. Furthermore, we highlight the functional characteristics of clinically significant miRNAs that play a crucial role in many cancers, including HNSCC. We also discuss the specific molecular mechanisms of miRNA regulation in an attempt to further unravel their implications in cancer initiation and progression. Finally, we outline how individual miRNAs and miRNA signatures can be used as novel biomarkers for the diagnosis, prognosis, and therapy of HNSCC, as well as their significance in the design and implementation of future therapeutic approaches.

## 2. The Role of the Most Common miRNAs in HNC

In this section, certain characteristics and biomarker abilities of the most commonly identified miRNAs in HNC and in other malignancies are thoroughly analyzed.

### 2.1. MiR-21

MiR-21 is currently one of the most extensively studied miRNAs, mainly because it is one of the few miRNAs that are found consistently overexpressed in a number of human cancer types, including ovarian [27], lung [28], gastric [29], breast [30], colorectal [31], B-cell lymphoma [32], and glioblastoma [33]. In a similar manner, miR-21 levels have been consistently found higher in the tumor tissue [34,35,36,37], serum (exosomes) [38,39], plasma [40,41,42,43], saliva [37], and whole blood [44] samples of patients, as compared to those of healthy controls, in the majority of HNC subtypes [40]. miR-21 has clear oncogenic properties in HNC, as it targets a number of known onco-suppressor genes, such as PTEN [45], p53 [46], p63 [47], and PDCD4 [48,49,50], thus playing an important role in several cancer-related processes, including cellular proliferation [51], invasion [51,52], metastasis [53], and apoptosis [48]. miR-21 has been proposed as a potential biomarker of diagnostic, prognostic, and therapeutic value in HNC. miR-21 expression has demonstrated high diagnostic accuracy in distinguishing between oral squamous cell carcinoma (OSCC) tumor tissue and healthy mucosa samples [54], and in evaluating cervical lymph node metastasis in patients with OSCC [37]. A number of different studies have reported that aberrantly increased levels of miR-21 expression correlate with advanced cancer stages, lymph node metastasis, poorer prognosis, and ultimately decreased survival of patients with laryngeal squamous cell carcinoma (LSCC) [34,55] and OSCC [56,57]. In the same context, miR-21 overexpression appears to be an independent prognostic marker of poor survival in patients with squamous cell carcinoma of the tongue (TSCC) [58]. miR-21 has also the potential to be used as a monitoring biomarker, since its expression levels seem to diminish after surgery in HNSCC patients with good prognosis, but remain high in patients with poor prognosis [36,41]. It has been suggested that through the targeting of PDCD4, miR-21 could sensitize chemoresistant TSCC to cisplatin treatment, thereby constituting a potential target for TSCC therapy [59]. 

### 2.2. miR-375

miR-375 has been found consistently downregulated in tumor tissue [60,61,62,63], serum [64], plasma [41,42], and saliva [63] samples of patients with HNC. miR-375 appears to play a tumor-suppressing role in HNC, as it has been shown to suppress cancer cell proliferation, migration, and invasion; this is possibly achieved by targeting XPR1 in esophageal squamous cell carcinoma (ESCC) [61], HNF1β in LSCC [65], as well as PDK1 [60] and USP1 [66] in nasopharyngeal carcinoma (NPC). In a study on patients with primary HNSCC, miR-375 expression was successfully utilized in distinguishing HNSCC carcinoma tissues from non-cancerous tumor-adjacent tissues with 87.5% sensitivity and 65% specificity [67]. The low expression of miR-375 was found to significantly correlate with cancer aggressiveness as it is associated with poor overall survival (OS) in ESCC [68] and higher TNM stages in LSCC patients [34]. These findings suggest that miR-375 could potentially serve as a diagnostic and prognostic marker in HNC. The downregulation of plasma miR-375 has been highly associated with disease recurrence after surgery in OSCC patients, suggesting that circulating miR-375 could be utilized as a post-operative surveillance marker for this type of HNSCC [42]. Different anti-cancer drugs (doxorubicin, 5-fluorouracil, trichostatin A, and etoposide) have been shown to reactivate and increase miR-375 expression in tongue cancer cells, possibly indicating that miR-375 may mediate cellular reactions in response to these drugs [69].

### 2.3. miR-99

The miR-99 family has been shown to function as a tumor suppressor in a number of cancer types, including cervical carcinoma [70], prostate cancer [71], and glioma [72]. In HNSCC, the overexpression of miR-99a and miR-100 suppresses cancer cell proliferation, migration, and invasion [73,74]. Similarly, miR-99*, a passenger strand of miR-99, appears to act as an onco-suppressor in HNC and its expression levels have been found to be significantly downregulated in the tumor tissues of patients with OSCC, compared to adjacent non-cancerous tissues [75,76]. miR-100, miR-99a, and miR-99b, also members of the miR-99 family, have all been found to be downregulated in the tumor tissue samples of HNSCC patients [73,77,78,79,80,81,82,83,84,85], with the exception of one study that reported miR-100 overexpression in the tumor tissue samples of HNSCC patients [86]. miR-99a and miR-100 expression has been found to be lower in the serum and plasma samples of OSCC patients [87,88], with miR-99a also appearing to be downregulated in the plasma samples of HNSCC patients [36], as compared to healthy controls. Notably, miR-99a expression levels have been found to be significantly higher in post-operative OSCC than in pre-operative samples, and have the potential to distinguish OSCC cases from healthy controls, indicating the potential of miR-99 as a diagnostic biomarker [87].

miR-99a, miR-99b, and miR-99* downregulation has been closely associated with LNM [73], advanced clinical stage [73,79,81,87], disease recurrence [75], and worse survival outcomes [89] in patients with HNSCC, while miR-99* overexpression has been shown to correlate with better progression-free survival (PFS) and overall survival (OS) [75]. Interestingly, increased miR-100 expression levels have been reported to significantly correlate with poorer survival outcomes, suggesting that miR-100 might be a prognostic biomarker in OSCC [79]. The aforementioned findings suggest that miR-99 members could be utilized as independent prognostic factors of OS in OSCC patients, and warrant additional follow-up studies to further assess and validate their prognostic value, as well as their potential as therapeutic markers.

### 2.4. miR-34a 

MiR-34a functions as a tumor suppressor and is downregulated in a number of cancer types [90], including colorectal [91], neuroblastoma [92], prostate [93], and thyroid cancer [94]. It interacts with a variety of genes that are known to be involved in oncogenic processes, such as p53 [95,96,97], Bcl-2 [98], cyclin D1 [99], CDK4 [90], and VEGF [90], and has been reported to affect tumor cell proliferation [90,99], apoptosis [95], senescence [100], invasion [98], metastasis [101], and drug resistance [102,103]. In HNSCC, expression levels of miR-34a appear to be significantly lower in tissue samples as compared to normal tissues, and for this reason miR-34a has been suggested as a novel and highly sensitive biomarker for diagnostic use in HNSCC [104]. In a number of other studies, similar expression patterns of miR-34a in HNC have been observed [105,106,107,108], with the exception of a study, comparing site-specific HNSCC samples, reported increased miR-34a expression levels in tumor tissues of oropharyngeal carcinoma [109]. Such findings suggest that miRNA expression profiles could be site-specific, consequently affecting the clinico-pathological features of patients with HNSCC [109]. miR-34a downregulation in NPC has been significantly correlated with bone metastasis and TNM staging, while low levels of miR-34a in NPC and sinonasal squamous cell carcinomas (sinonasal SCC) patients have been correlated with a decreased 5-year survival rate [110,111]. These findings suggest that miR-34a expression could serve as a marker for disease prognosis.

### 2.5. Let-7

Most members of the let-7 family have been found downregulated in the tumor tissues [112,113,114], serum [115,116], and saliva [117] of HNSCC patients, although several studies have also reported an upregulation of certain let-7 members in the tumor tissues and serum of HNSCC patients [115,116,118]. Let-7 miRNAs have been characterized as tumor suppressors in HNC, targeting a number of important oncogenes, including K-RAS [119] and HMGA2 [120]. A number of let-7 family miRNAs have been linked to the regulation of different molecular pathways that contribute to several oncogenic properties of tumor cells, including invasion [120], metastasis [120], stem-like properties [121], EMT [114], and chemoresistance [114].

Salivary let-7a expression levels have demonstrated high sensitivity and specificity in differentiating between HNSCC patients and healthy controls [117]. In addition, let-7a levels have been reported to correlate with different clinical stages of the disease, since lower let-7a expression was observed in advanced laryngeal cancer compared to early-stage samples [120]. Similarly, low levels of let-7 family miRNAs in HNSCC tumors have been significantly associated with worse survival outcomes and more aggressive forms of cancer, suggesting that let-7 could be a potential prognostic marker [112,122]. 

With regards to therapeutic applications, let-7 appears to be a particularly prospective target, as several studies have reported that the overexpression or restoration of let-7 levels in tumor cells could suppress different oncogenic characteristics that might enhance treatment outcomes [113,123,124]. 

### 2.6. MiR-200

The miR-200 family consists of five members (miR-200a, -200b, -200c, -141, -429), which are grouped into two independent transcriptional clusters (the first one containing miR-200a, miR-200b and miR-429, and the second containing miR-200c and miR-141) [125,126,127]. All miR-200 members have been shown to play an important part in repressing malignant cell transformation and in inhibiting tumor initiation, and have thus been characterized as tumor suppressors [127,128]. More specifically, the miR-200 family has been reported to negatively modulate the progress of EMT in various cancers, including HNSCC [129], by controlling the expression of transcriptional repressors ZEB1 and ZEB2, the so-called EMT master regulators [126,130,131].

In terms of predictive and prognostic applications in the clinical setting, a number of studies have highlighted the miR-200 family as promising potential targets. MiR-200c and miR-141 expression levels appear to be significantly downregulated in HNSCC tumor tissues, as compared to non-cancerous samples [126,132]. MiR-200a is present at significantly lower levels in the saliva samples of OSCC patients, as well as in HNSCC patients before radiotherapy [133], suggesting that miR-200a has the potential to serve as a novel, non-invasive marker of detection and monitoring [133,134]. Furthermore, downregulated miR-200a and miR-200c have demonstrated a significant correlation with disease recurrence in LSCC patients after surgery [130].

Several studies also hint at the therapeutic potential of miR-200 family members. In particular, it has been shown that the induced expression of miR-200 family members successfully inhibits the metastatic ability and malignant cancer stem cell (CSC)-like properties of HNSCC cells [132,135,136]. These findings suggest that the restoration of miR-200c levels could be a promising step towards more successful treatment of advanced malignant HNSCC or HNSCC-derived CSC populations [132].

### 2.7. MiR-31

The literature on miR-31 is quite controversial, as this particular miRNA appears to be downregulated in certain types of cancer, such as liver [137], prostate [138], and triple negative breast cancer [139], but upregulated in colorectal [140], cervical [141], rectal [142], and certain subtypes of lung cancer [143], suggesting that the mode of miR-31 regulation depends on cancer histology. Overall, in HNC, miR-31 mostly appears to be upregulated [46,47,79,144,145,146], acting as an oncogene and promoting the proliferation and invasion of tumor cells [147]. However, contradicting results have even been reported among different HNC subtypes. In a comparative study, it was shown that miR-31 levels in plasma samples were significantly higher in OSCC patients, as compared to healthy controls [148]. Similarly, miR-31 expression was found to be upregulated in the tissue samples of HNSCC patients [149,150]; it has therefore been proposed that particular miRNAs may promote the development of HNSCC by suppressing the FIH-mediated activation of HIF [149], a gene that promotes angiogenesis. On the other hand, two recent studies reported significantly lower miR-31 expression levels in the peripheral blood samples of patients with NPC, as compared to the healthy controls [151], and a downregulation of salivary miR-31 in oropharynx squamous cell carcinoma (OPSCC) patients, as compared to controls [152], respectively. Furthermore, the downregulated expression of miR-31 has been observed in LSCC tissues, and has been correlated with advanced stages of the disease [153]. These findings suggest that miR-31 expression levels are most likely tumor site-specific and might even vary among different sample types. Particularly, it has been shown that miR-31 is more abundantly expressed in the saliva than in the plasma of OSCC patients, thereby suggesting that the salivary detection of miR-31 could represent a more sensitive marker for the diagnosis of oral malignancy [154].

The utility of miR-31 as a biomarker is further explored in several studies which suggest that, in various HNSCC patient sample types (saliva, plasma, tumor tissues), the expression levels of miR-31 increase and are positively correlated with poor pathological parameters [147,153] and advanced staging [155]. MiR-31 quantification could also serve as a useful marker of post-operative follow-up of OSCC, as salivary levels appear to be remarkably reduced after the excision of the oral carcinoma [154].

### 2.8. MiR-125a/miR-125b 

The miR-125 family, composed of the four homologs, miR-125a-3p, miR-125a-5p, miR-125b-1, and miR-125b-2, has been found to play an important role in a number of cancer types, acting either as promoters or suppressors of tumorigenesis [156]. In HNC, these miRNAs are mainly considered to be tumor suppressors and have been found to be significantly downregulated in the tumor tissue [47,157,158,159,160,161,162], plasma [163], and saliva [133,134] samples of patients; however, the precise functional role and mechanism of action related to cancer progression remain quite unresolved. Although miR-125b-2-3p has been reported to suppress tumor progression, LNM, and distant metastasis in HNSCC [83], in another study, miR-125b levels appear to be significantly higher in the metastatic primary HNSCC tumor samples, as well as in the respective metastatic tumor itself, compared with non-metastatic tumors [164], highlighting the controversial role of this miRNA in HNSCC. It is noteworthy that while these findings suggest a strong potential role for miR-125b as a biomarker of metastases and lower disease-specific survival, it is also indicated that miRΝA expression patterns can shift between initial and later stages as the disease progresses [164]. Last but not least, miR-125b has been proposed as a prognostic marker for OSCC, as its downregulation seems to correlate with tumor stage [165], radioresistance [165], poor prognosis, and worse patient survival [162], while miR-125a-5p has been suggested as an individualized biomarker, as its downregulation has been associated with locoregional recurrence and an overall poor prognosis [166]. 

### 2.9. MiR-196a/miR-196b

The miR-196 family, which includes two mature miRNA members, miR-196a and miR-196b, has been found to be aberrantly expressed in a number of cancers [167]. MiR-196a and miR-196b seem to be consistently upregulated in the tumor tissue [57,168,169], plasma [170,171,172], and saliva [168] samples of HNC patients as compared to corresponding healthy controls. Both miRNAs possess oncogenic properties. The overexpression of miR-196a has been shown to significantly increase cell proliferation, migration, and invasion, and to induce EMT, possibly through targeting of ANXA1 [173] and MAMDC2 [174] in HNSCC cells, and of HOXB8 and p27 (CDKN1B) in OSCC cells [175]. MiR-196b has been demonstrated to promote cell proliferation and invasion, and to suppress apoptosis by targeting SOCS2 [176] and PCDH-17 [177] in LSCC cells and ANXA1 in HNSCC cells [178]. The combined detection of plasma miR-196a and miR-196b expression appears highly potent in diagnosing oral cancer patients with high sensitivity and specificity [171]. The upregulation of miR-196a has been shown to strongly correlate with poor prognosis and worse survival outcomes in OSCC patients [57,170,172] and LNM in TSCC patients [179]. It has also been highlighted as a promising marker of HNSCC response to radiotherapy, since miR-196a overexpression appears to increase radioresistance in HNSCC cells [173]. Notably, both miR-196a and miR-196b have been characterized as potentially important therapeutic targets, since the inhibition of these miRNAs has been shown to reduce cell proliferation in oral cancer and LSCC cell lines [175,177,180]. Furthermore, both miR-196a and miR-196b have been suggested as promising diagnostic, prognostic, and therapeutic biomarkers for LC. High miR-196b expression levels have been shown to correlate with worse clinicopathological parameters in patients with LSCC; for this reason, it has been suggested as an independent prognostic factor of OS in LSCC patients [176].

### 2.10. MiR-9

MiR-9 (miR-9-5p and miR-9-3p) is a miRNA that is very often deregulated in cancer; however, its biological role has proven to be quite complex, as it can behave as an oncomiR in certain cases, and as an onco-suppressor miRNA in other cases [181,182]. MiR-9 appears to be downregulated in gastric [183], colorectal [184], and hepatocellular carcinoma [185], and advanced non-small cell lung cancer [186], but upregulated in breast [187] and cervical cancer [188]. In HNC, the regulation and functional role of miR-9 seem to vary, and might depend on the anatomic site or cellular context. For example, miR-9 levels have also been found overexpressed in the tumor tissue samples of sinonasal SCC [189] and in the saliva samples of HNSCC patients, as compared to corresponding healthy controls [190,191]. However, miR-9 is downregulated in the tumor tissue [192] and plasma samples of NPC patients [193,194], and in the tumor tissue [195,196] and serum samples of OSCC patients [197], which is associated with various clinicopathological parameters and poor OS and disease-free survival (DFS); this suggests that miR-9 downregulation in serum could represent a promising independent prognostic factor for OSCC [197].

Interestingly, a higher expression of miR-9-3p has been shown to correlate with the presence of vascular and perineural invasion, whereas the overexpression of miR-9-5p has been associated with longer survival in sinonasal SCC patients [189]. A recent study has also proposed that miR-9 may represent a valuable predictive biomarker of the response to radio-chemotherapy, since high miR-9 expression appeared to be associated with poor prognosis in HNSCC patients treated with RT+CTX (radiotherapy combined with the anti-EGFR monoclonal antibody cetuximab) [198].

### 2.11. MiR-181a

The miR-181 family is a group of highly conserved miRNAs that act as regulators in various physiological functions, including proliferation, apoptosis, autophagy, mitochondrial activity, and immune response [199,200,201]. The miR-181 family is differentially expressed in solid tumors and hematological cancers and may act either as cancer cell inhibitors or as cancer promoters, underlining diagnostic and prognostic significance [202]. Four mature forms of the miR-181 family are present in the human genome, including miR-181a, miR-181b, miR-181c, and miR-181d.

miR-181a is normally expressed in the neurons, blood, and lymph nodes. In various cancers, aberrant miR-181a expression causes alterations in the expression patterns of genes, leading to tumor progression and cancer cell migration [202]. In particular, abnormal miR-181a expression has been linked to colorectal [203,204], non-small cell lung [205], pancreatic [206], ovarian [207], and prostate cancer [208], as well as HNSCC [209]. In this context, it has been suggested that laryngeal carcinoma cell invasion and migration is inhibited by miR-181a upregulation, as the latter appears to regulate metastasis-related genes, such as N- and E-cadherin and ATF2 [210].

On the other hand, in OSCC patients, miR-181a plasma levels appear to be aberrantly increased and correlate with advanced LNM and vascular invasion, identifying miR-181a as a valuable non-invasive prognostic biomarker [211]. Interestingly, HNC patients, who are also positive for HPV infection, have decreased levels of miR-181a, as expression is suppressed by HPV subtype 16 [212,213]. The downregulation of miR-181a in plasma could represent a useful biomarker of early disease detection in esophageal cancer patients, and possibly for the follow-up of patients, based on evidence that post-operative plasma miR-181a levels are significantly increased [214]. Similarly, miR-181a expression appears to be increased in the serum of ESCC patients, who respond to radiotherapy [215]. This suggests that serum miR-181a quantification could represent a minimally invasive, predictive marker of response to radiotherapy in ESCC patients. However, miR-181a overexpression in tissue correlates with advanced TNM stages and the detection of LNM; therefore, it can be associated with the development or pathogenesis of ESCC [216].

### 2.12. MiR-155 

miR-155 is known to be implicated in the development of several types of cancer, such as non-small cell lung cancer [217], breast [218], gliοma [219], colorectal [220], and HNSCC [221]. In addition, miR-155 expression levels are increased and correlated with patient survival in hepatocellular carcinoma [222]. Increasing evidence suggests that miR-155 is aberrantly expressed in HNC, indicating a strong correlation with patient survival and the metastatic activity of cancer cells. Specifically, serum and plasma miR-155 levels are upregulated in HNSCC patients [64,223] and circulating miR-155 is characterized as a non-invasive HNSCC biomarker, since patients with higher levels of miR-155 also have a higher risk of relapse [64]. In this context, miR-155 may play a role as an oncogenic miRNA in ESCC and OSCC. Indeed, samples derived from tumor tissues have significantly higher levels of miR-155 [160,224,225,226,227], whereas miR-155 overexpression is highly associated with ESCC tumor aggressiveness [224] and LNM and disease recurrence in OSCC patients, indicating that it could be used as a prognostic biomarker for survival [225,226]. The increased expression of miR-155 in HNSCC tissues is probably associated with immune cell tumor infiltration, since the upregulation of the miR-146a/miR-155 cluster has been highly correlated with the expression of immune cell-related mRNA, including NK cells, B-cells, T regulatory cells (Treg), Th1 cells, CD8+ T-cells, and activated CD4+ T-cells [228]. The increased mRNA expression of these immune cell types has also been significantly associated with prolonged OS after the combined administration of chemo- and radiotherapy [228]. miR-155 is also overexpressed in cultured HNSCC cells, resulting in increased cell proliferation, invasion, and decreased apoptotic activity, via the downregulation of the CDC73 gene, which normally favors the inhibition of cell growth and proliferation [229]. Interestingly, certain studies have also shown a reduction in miR-155 levels in the peripheral blood of HNSCC patients [230]. The downregulation of miR-155 increases the risk of distant metastasis and is correlated with the size and extent of the tumor, suggesting that its expression is also related to the survival of TSCC patients [231]. Such observations also suggest that the type of affected tissue and the tumor site can modify the expression levels of particular miRNAs, including miR-155, implying that distinct tissue- and site-specific miRNA profiles are present in HNC.

### 2.13. MiR-146a

miR-146a is an abundantly expressed miRNA, involved in physiological processes such as the regulation of the immune system, cell maturation, differentiation, and inflammatory responses [232]. The aberrant expression of miR-146a has been detected in several human malignancies, including B cell lymphomas [32], lung [233,234], breast [235], gastric [236], cervical [237], hepatocellular [238], prostate [239], colorectal [240], glioma cancers [241]. miR-146a has been proposed as a marker of HNSCC in both the tumor site and in circulation; miR-146a overexpression is present in tumor tissues, as compared to neighboring non-cancerous tissue in OSCC and LSCC [7]. By evaluating the levels of miR-146a in oral cancer patients and healthy controls, the plasma miR-146a level was found to be higher in the study group [242]. Interestingly, post-operative miR-146a levels are significantly reduced in oral cancer patients, possibly indicating that miRNAs detected in the circulation might originate from tumor cells [242]. In ESCC, miR-146a is downregulated in tumor tissues and serum and is associated with an increase in TNM stage [243,244]. These observations suggest that miRNA expression highly depends on tissue type, as well as on tumor location.

miR-146a could be characterized as a biomarker of prognosis for distant metastasis, since its expression in the whole blood of HNSSC patients has been negatively correlated with the presence of secondary tumors [230]. The expression profile of miR-146a in HNSCC patients is also correlated with human papillomavirus (HPV) infection, with HPV + HNSCC patients exhibiting significantly higher tumor miR-146a levels, as compared to non-infected patients [245]. Moreover, the evaluation of miRNA levels in the peripheral blood mononuclear cells (PBMCs) of ESCC patients has indicated an upregulation of miR-146a, which appears to be a major component of the miR-146a-IRAK1-NF-κB axis [246].

### 2.14. MiR-23a

miR-23a belongs to the miR-23a-27a-24-2 cluster and is aberrantly expressed in several malignancies, mainly serving as a biomarker for cancer detection [247,248]. miR-23a is overexpressed in breast [249], gastric [250], hepatocellular carcinoma [251], lung [252], pancreatic and colorectal [253], and ovarian cancers [254], as well as acute myeloid leukemia [255]. The biological role of miR-23a in cancer is highly controversial. MiR-23a has been reported to negatively regulate cancer cell development and metabolic activity [256], and the increased expression of miR-23a stimulates caspase-induced apoptosis [257]. It is also suggested that miR-23a overexpression contributes to enhanced chemosensitivity in hepatocellular carcinoma cell lines [258]. In line with this, miR-23a expression has also been correlated with HNC; specifically, miR-23a negatively regulates glucose metabolism and ATP production in HNSCC cell lines and therefore inhibits cancer cell growth and proliferation [256]. On the contrary, miR-23a has been shown to enhance viability, proliferation, and mobility of renal cancer cell lines [259], while contributing to increased chemoresistance in TSCC [260]. In the tissue samples of ESCC patients, miR-23a is upregulated [261,262] and is associated with advanced TNM staging and the development of LNM, leading to a more aggressive cancer phenotype, thus indicating that the expression patterns of this particular miRNA could affect ESCC progression [261]. The miR-23a is also aberrantly overexpressed in the serum of ESCC patients that exhibit lower response rates to neoadjuvant chemotherapy [263]. Interestingly, the quantification of the serum expression levels of three combined miRNAs, namely of miR-193b, miR-873, and miR-23a, could facilitate the identification of responders to neoadjuvant chemotherapy, thereby representing a non-invasive predictive signature of response to therapy in ESCC patients [263].

ESCC patients with low rates of response to chemotherapy present with significantly higher miR-23a tissue and plasma expression levels, with the latter also being significantly correlated with increased TNM staging and lymph node invasion [264]. This highlights a role for the evaluation of miR-23a levels in the circulation and the tumor samples of ESCC patients for their appropriate stratification based on cancer progression and response to treatment. The inhibition of miR-23a leads to decreased therapeutic resistance in TSCC cells, indicating a possible role for miR-23a as a biomarker for the appropriate stratification of HNSCC patients and the prediction of response to chemotherapy [265].

### 2.15. MiR-16

miR-16 used to serve as an internal control and a miRNA reference for the normalization of other miRNAs. However, it was suggested that miR-16 is abundantly expressed [266] and highly conserved as one of the first miRNAs to be linked to human cancers [267]. miR-16 is a central regulator of the cell cycle, suppressing cell proliferation and tumorigenicity both in vitro and in vivo and promoting cell apoptosis [268]. It has also been well documented as a prominent tumor suppressor in several types of malignancy, including breast [39], colorectal [269], bladder [270], prostate [271], and non-small lung cancer [272]. In HNSCC, miR-16 can be downregulated or upregulated, depending on the cancer subtype and the tissue in which it is expressed. In OSCC tissues, miR-16 is markedly decreased compared to adjacent non-cancerous tissues [273,274,275]. Salivary miR-16 levels are also downregulated in patients with OSCC [276]. Notably, miR-16 expression is downregulated in patients with metastatic oral cancer and a more advanced TNM stage [162,273], and it is associated with poor overall survival; therefore, it could be used as a robust prognostic biomarker [162].

In NPC patients, plasma miR-16 levels are significantly higher compared to controls, indicating a negative correlation with NPC progression [43]. In ESCC, miR-16 has been characterized as an oncogenic miRNA, significantly upregulated in the serum and tumor samples of patients; the high correlation of aberrant miR-16 expression with LNM and increased tumor size seems to contribute to a decreased OS and DFS in these patients and warrants further investigation into its potential to be used as a prognostic biomarker [277,278,279].

### 2.16. MiR-29

The miR-29 family includes three miRNAs, namely miR-29a, miR-29b, and miR-29c [280]. Several studies have demonstrated that the miR-29 family is mostly downregulated in cancer. Low miR-29 expression has been observed in several solid tumors, such as glioblastoma [281], hepatic cholangiocarcinoma [282], prostate [283], ovarian [284], endometrial [285], non-small cell lung cancer [286], HNC [287], as well as hematopoietic malignancies [288,289]. On the contrary, the miR-29 family appears to be upregulated in breast cancer [290], suggesting that the mode of miR-29 expression probably depends on the cancer type. In general, however, miR-29 family members are regarded as tumor suppressors and usually function as negative regulators of oncogenes or as enhancers of tumor suppressor genes [280]. Specifically, it has been observed that miR-29 promotes cancer cell apoptosis and inhibits tumor cell proliferation and invasiveness by suppressing EMT activity [280].

miR-29 expression levels are significantly decreased in HNSCC tissues and cell lines, as compared to adjacent non-cancerous samples [291,292]. The induced expression of miR-29 hampers the ability of HNSCC cells to proliferate and migrate [291]. In this context, the miR-29 family could be classified as a miRNA tumor-suppressive signature in HNSCC, achieved via targeting LAMC2 and its ligand ITGA6 [291].

miR-29 is a significantly downregulated LC tumor, compared to adjacent normal tissues [293], as well as in NPC tissue samples and cell lines [294]. It is suggested that miR-29c targets the TIAM1 gene, which is closely related to the metastatic activity of cancer cells [294]. In TSCC, miR-29b functions as a tumor suppressor of miRNA, as it is significantly downregulated in both patient tissues and cell lines [295].

There are also references that correlate miR-29 expression levels with the outcome of radiotherapy in HNC patients. Guo et al. (2019) suggested that miR-29a is significantly downregulated in radioresistant NPC patients and cell lines, while the induced expression of miR-29a results in decreased cell viability and enhanced apoptotic activity following cell irradiation [296]. On the same note, miR-29b has also been found to regulate radiosensitivity in HNSCC. In particular, serum miR-29b levels appear to be significantly decreased in ESCC patients with radioresistance [297]. Overall, the aforementioned studies suggest that the miR-29 family act as tumor-suppressive miRNAs, through the regulation of genes and pathways that are involved in the cell cycle and metastasis of HNSCC cells, and could constitute a promising therapeutic target for HNSCC and related subtypes. Moreover, there is enough evidence to suggest that miR-29 could be used as a prognostic biomarker of improved radiotherapeutic outcomes [296,297]. Currently, there are two clinical trials studying the prognostic and therapeutic significance of the miR-29 family in HNC (discussed in a relevant section of this review).

**Figure 1:**
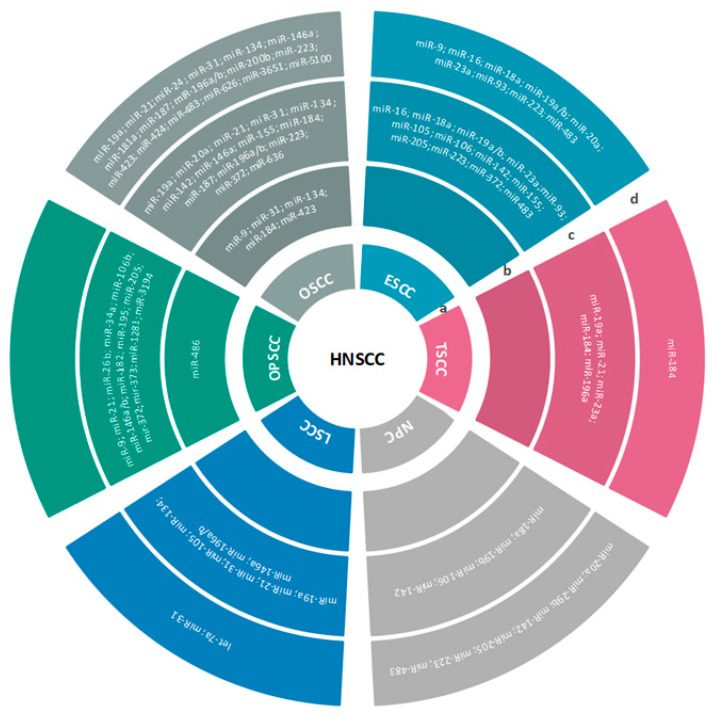
Overview of the most significant miRNAs that are upregulated in HNSCC. The first inner ring represents the subtypes of HNSCC (**a**); the second, third, and fourth rings represent miRNAs detected in saliva (**b**); tumor tissues (**c**); serum, plasma, or peripheral blood (**d**) of HNSCC patients.

**Figure 2:**
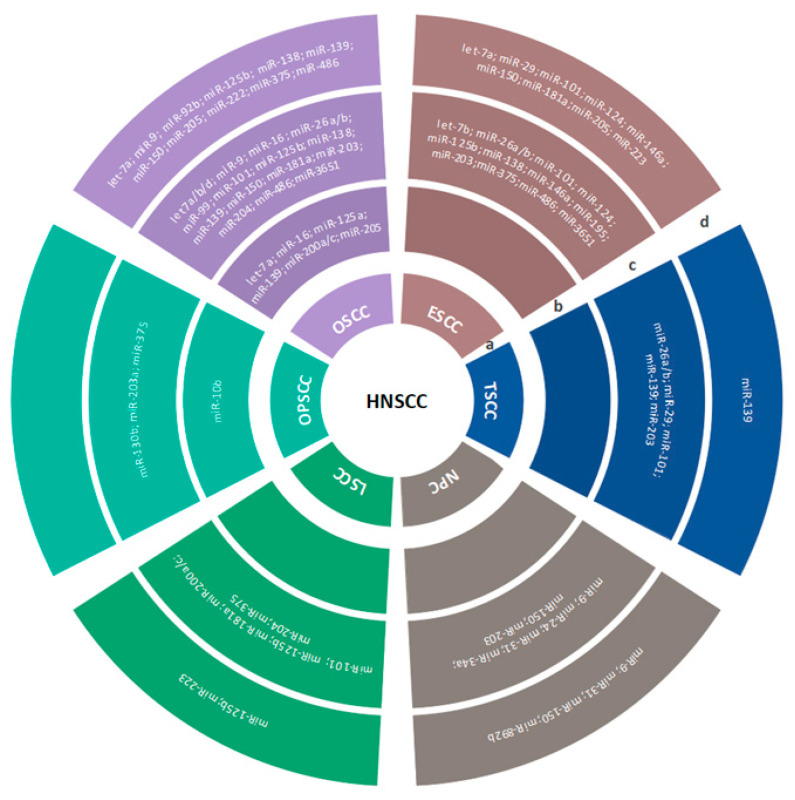
Overview of the most significant miRNAs that are downregulated in HNSCC. The first inner ring represents the subtypes of HNSCC (**a**); the second, third, and fourth rings represent miRNAs detected in saliva (**b**); tumor tissues (**c**); serum, plasma, or peripheral blood (**d**) of HNSCC patients.

The most commonly upregulated and downregulated miRNAs that have been reported as potential biomarkers in HNSCC are presented in Table 1, Figure 1, Table 2, and Figure 2, respectively. The identified gene hotspots and/or respective pathways and cellular functions targeted by miRNAs in HNC are presented in Table 3.

## 3. MiRNA Signatures of Diagnostic, Prognostic, and/or Predictive Value in HNC

Although it has been well established that aberrant miRNA expression could serve as a potential biomarker in cancer patients, it should be noted that miRNA deregulation has also been observed in other pathological conditions. For example, abnormally high levels of miR-21, one of the most extensively studied and upregulated miRNAs in HNC, have also been reported in several non-cancerous pathologies, including cardiorenal syndrome [421], restrictive allograft syndrome (RAS) in lung transplant recipients [422], traumatic brain injury [423], and sepsis-associated cardiac dysfunction [424,425]. This is not entirely surprising since miRNAs are well known for targeting multiple genes and for regulating different cellular functions; it does, however, highlight that miRNA might not constitute cancer-specific biomarkers and that single miRNA expression profiles might not be sufficient for diagnostic and prognostic use. In order to increase the predictive properties of miRNA alterations, recent studies have turned to investigating the expression patterns and ratios of multiple miRNAs combined together as opposed to individual miRNA expression profiles.

### 3.1. MiRNA Ratios/miRNA Combined Expression

The miR-221 to miR-375 expression ratio has been shown to differentiate between normal tissue and HNC, with 92% sensitivity and 93% specificity, and could therefore be reflective of disease status [62]. In addition, the expression ratio of miR-196a to miR-204 appears to be a very strong predictor of disease recurrence and survival outcome in patients with OSCC, exhibiting 91% specificity and 83% sensitivity in distinguishing aggressive from non-aggressive tumors [57]. In patients with LSCC, the expression ratio of miR-21 and miR-375 has demonstrated 94% sensitivity and specificity in disease detection [55], whereas a high miR-21/miR-375 expression ratio has also been proposed as a prognostic biomarker of a higher tumor stage and worse prognostic outcomes [426]. Similarly, combined differential expression of miR-21 and miR-375 has been demonstrated to accurately predict TSCC both in oral cytology (100% sensitivity and 64% specificity) and in tissue samples (83% sensitivity and 83% specificity), with the former representing a potential non-invasive tool for early diagnosis and possibly for TSCC screening in the future [49]. Additionally, the combined differential expression of miR-6510-3p and miR-34c-5p appears quite promising in distinguishing between healthy tissue and OSCC with 94.6% specificity and 91.9% sensitivity, while the combined expression of miR-449a-5p, miR-6510-3p, and miR-133a-5p seems to differentiate between healthy tissue and LSCC samples with 87.9% specificity and 90.9% sensitivity [7].

### 3.2. Multiple miRNA Signatures

#### 3.2.1. HNSCC

A 3-miRNA serum signature, consisting of miR-383, miR-615, and miR-877, has shown excellent diagnostic potential in differentiating between patients with HSNCC and healthy controls, with 89.3% sensitivity and 98.9% specificity [277]. Another non-invasive saliva-based panel of three miRNAs, consisting of miR-9, miR-134 and miR-191, has been proposed as a reliable marker for the diagnosis of HNSCC [191], while an 8-miRNA circulating plasma signature, consisting of miR-21-5p, miR-28-3p, miR-142-3p, miR-191-5p, miR-186-5p, miR-197-3p, miR-425-5p, and miR-590-5p, has also demonstrated good performance in distinguishing between HNSCC patients and healthy controls [331].

**Figure 3:**
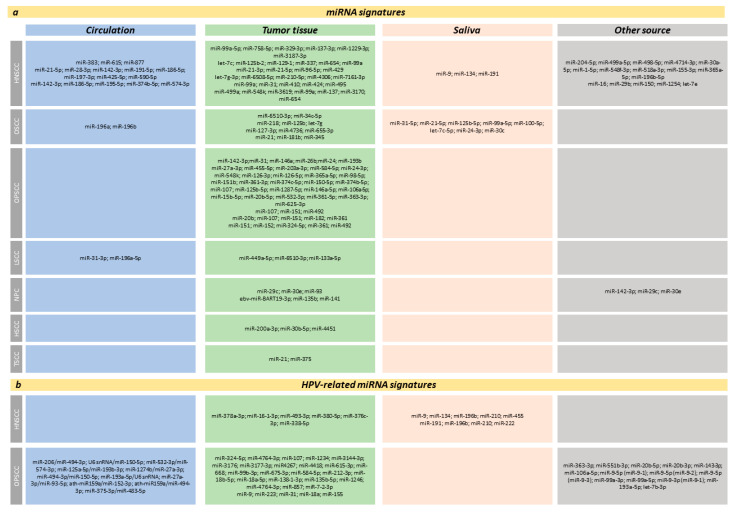
(**a**) Overview of miRNAs signatures in HNSCC subtypes; (**b**) miRNA signatures detected in HPV+ HNSCC and OPSCC patients. The miRNA signatures are detected and evaluated in the circulation (serum and/or plasma), tumor tissues, and saliva of HNSCC patients and in vitro, in HNSCC cell lines. The term other source refers to data derived from literature and bioinformatic analyses.

An 11-miRNA signature (Table 4 and Figure 3a) [24] and a 6-miRNA signature (Table 4 and Figure 3a), and their respective nomogram-based models, have shown great potential in accurately predicting 3- and 5-year survival outcomes in HNSCC patients [427]. Similarly, a 6-miRNA signature (let-7c, miR-125b-2, miR-129-1, miR-337, miR-654, and miR-99a) has been identified as an independent predictor of HNSCC patient survival, as demonstrated in a large The Cancer Genome Atlas dataset (TCGA) [428]. Another signature, composed of four known upregulated miRNAs (miR-21-3p, miR-21-5p, miR-96-5p, and miR-429), when detected in the peritumoral tissue of HNSCC patients, has been associated with shorter local recurrence-free survival and could therefore represent an independent predictive marker of patients at high risk for disease recurrence [429]. On the other hand, a 5-miRNA signature (Table 4 and Figure 3a) has been identified as an independent predictor of disease recurrence (post-radio/chemotherapy) and survival in HPV-negative HNSCC patients [430]. When combined with clinical parameters (TNM stages and extracapsular extension), this same signature appears useful in further categorizing HNSCC patients into distinct risk groups for recurrence, thereby presenting a promising marker for patient stratification in personalized treatment [430]. The combined upregulation of a different 5-miRNA group in the plasma of HNSCC patients, namely miR-142-3p, miR-186-5p, miR-195-5p, miR-374b-5p, and miR-574-3p, has demonstrated significant correlation with poor prognostic outcomes in patients treated with radiochemotherapy [331], while a prognostic model of seven miRNAs (Table 4 and Figure 3a) has shown good specificity and sensitivity in predicting OS in HNSCC patients and in successfully distinguishing between low- and high-risk patient groups, which could potentially improve patient management in the near future [431]. A number of miRNAs have also been associated with patient response to radiotherapy. Two different 5-miRNA signatures (the first consisting of miR-16, miR-29b, miR-150, miR-1254, and let-7e [432] and the second of miR-99a, miR-31, miR-410, miR-424, and miR-495 [433]) have been proposed as markers for predicting radiation responsiveness in HNSCC and could potentially aid in the optimization of radiation strategies.

#### 3.2.2. OSCC

A panel of eight frequently deregulated miRNAs in OSCC, including miR-31-5p, miR-21-5p, miR-125b-5p, miR-99a-5p, miR-100-5p, let-7c-5p, miR-24-3p, and miR-30c, has demonstrated high accuracy in detecting the presence of cancer cells in salivary samples, exhibiting high specificity (100%) in identifying OSCC cases (15/15) [434]. The expression signature of miR-21, miR-181b, and miR-345 has been associated with disease progression in OSCC, as it has been shown to successfully differentiate progressive leukoplakia/OSCC from non-progressive leukoplakias/normal tissue [437]. A combined differential expression of miR-218, miR-125b, and let-7g has in turn been proposed as an important prognostic indicator in patients with OSCC, as it has demonstrated significant association with DFS and disease-specific survival (DSS) [435]. Furthermore, a miRNA-based 5-plex marker panel, consisting of miR-127-3p, miR-4736, miR-655-3p, TNM stage, and histologic grading, has been found useful in assessing the prognostic status in early-stage OSCC patients [436].

#### 3.2.3. OPSCC

A combined expression signature of 6 miRNAs (miR-142-3p, miR-31, miR-146a, miR-26b, miR-24 and miR-193b) appears to have prognostic significance in OPSCC [438]. Similarly, an miRNA-seq profiling analysis has identified a 26-miRNA signature (Table 4 and Figure 3a) that proved highly useful in distinguishing between high- and low-risk HPV-positive patients with OPSCC, further suggesting that the particular signature could potentially improve the HPV-positive OPSCC patient selection for personalized therapies [439]. Three additional groups of miRNAs have been significantly associated with different clinicopathological features in OPSCC patients, including OS (miR-107, miR-151, miR-492), DFS (miR-20b, miR-107, miR-151, miR-182, miR-361), and distant metastasis (miR-151, miR-152, miR-324-5p, miR-361, miR-492) [440].

#### 3.2.4. HSCC/NPC

A 3-miRNA signature, consisting of miR-200a-3p, miR-30b-5p, and miR-4451, has been proposed as a prognostic biomarker for post-operative HSCC patients treated with radiotherapy, since individuals with miR-200a-3p, miR-30b-5p, and miR-4451 upregulation have worse OS and DSS compared to those with lower miRNA expression levels [441]. 

Several signatures have also been identified as candidate biomarkers for NPC. The miR-29c, miR-30e, and miR-93 expression signature has been suggested as a reliable independent prognostic marker for NPC patients [442], while two different 3-miRNA signatures, the first one consisting of ebv-miR-BART19-3p, miR-135b, and miR-141 [443] and the second entailing miR-142-3p, miR-29c, and miR-30e [334], could be potentially used to predict OS in patients with NPC.

## 4. Dynamic Correlation between miRNAs and HPV Status in HNSCC

Human papillomavirus (HPV16, HPV18) infection is one of the major risk factors for developing OPSCC, along with smoking and alcohol consumption [366,444]. HPV-positive OPSCC, and its subtype, TSCC, are considered a distinct sub-group of HNSCC, presenting entirely different epidemiological, biological, histopathological, clinical, and molecular characteristics from HPV-negative OPSCC [445,446,447]. Although the true extent of HPV implication in HNSCC is not yet clear [448], HPV presence is a favorable prognostic factor for OPSCC patients, as it is associated with better response to radio-chemotherapy and higher OS [366,446]. Despite their differential disease profiles, both HPV-positive and HPV-negative OPSCC patients usually receive the same treatment (chemotherapy or chemoradiotherapy), meaning that most HPV-positive OPSCC patients are likely overtreated, while patients who belong in the poor prognosis group still present low survival outcomes [446,447]. This highlights the urgent need to identify and establish suitable biomarkers for the early detection of HPV-positive OPSCC cases, as well as for patient selection for personalized treatment options.

MiRNAs could potentially fulfill this need, since the presence of HPV in HNSCC has been shown to correlate with specific miRNA expression profiles that differ from those associated with HPV-negative HNSCC [145,446,449,450]; nonetheless, the studies that correlate the HPV status with the expression levels of particular miRNAs are still quite limited and the results are often controversial. A comparative study between HPV-positive and HPV-negative tonsillar tumors has identified 30 miRNAs exclusively expressed in HPV-positive tonsillar tumors, as well as a core of five miRNAs (miR-141-3p, miR-15b-5p, miR-200a-3p, miR-302c-3p, and miR-9-5p), most commonly found in HPV-positive cancers, including TSCC [451]. MiR-1281 and miR-3194-5p have been found significantly upregulated in HPV-positive primary TSCC compared to HPV-negative tumor tissues [366], while high expression levels of miR-155 have been associated with HPV-positive status in both tonsillar and base of tongue squamous cell carcinoma (BOTSCC) tissues [231]. MiR-99a-3p, miR-411-5p, and miR-4746-5p also appear de-regulated in HPV-positive HNSCC tumor samples, with miR-99a-3p and miR-4746-5p displaying significant upregulation and miR-411-5p equivalent downregulation [452]. Pathway enrichment analysis suggested that these particular miRNAs could be associated with HNSCC progression, as well as patient prognosis [452]. A microarray analysis has identified a total of 17 differentially expressed miRNAs between HPV-positive and HPV-negative TSCC tissues; however, when all tumor sub-sites were included together in one group, only five of those miRNAs (miR-16-3p, miR-29a, miR-29c, miR-150, and miR-363) showed statistically significant differential expression between HPV-positive and HPV-negative HNSCC tumors [245]. Another combination of five miRNAs (miR-16-3p, miR-20b, miR-142-3p, miR-150, and miR-363) accordingly displayed statistical significance between HPV-positive and HPV-negative tumors of exclusively oropharyngeal origin [245]. An additional combination of miRNAs, including miR-363, miR-33, miR-155, miR-181a, miR-181b, miR-29a, miR-218, miR-222, miR-221, and miR-142-5p, has been reported to be differentially expressed between HPV-positive and HPV-negative HNSCC cell lines [449]. Another study has identified a total of 36 differentially expressed miRNAs between HPV-positive and HPV-negative tonsillar squamous cell carcinoma samples, along with a different 14-miRNA core (miR-10b, miR-15a, miR-16, miR-20b, miR-139-3p, miR-139-5p, miR-145, miR-199a-3p, miR-199a-5p, miR-199b-5p, miR-328, miR-379, miR-381, and miR-574-3p) of HPV-positive HNSCC and cervical squamous cell carcinoma (CSCC) [448]. Among these miRNAs, the miR-15a/miR-16/miR-195/miR-497 family, the miR-106-363 cluster, and miR-143/miR-145 seem to have specific target genes associated with HPV pathogenesis, but most of them have never been analyzed in the context of HNSCC, since HPV status is rarely considered; thus, more functional in vitro research is warranted in the future to validate these findings [448].

Of all the miRNAs identified in the aforementioned studies, miR-9 appears to be one of the most frequently associated with HPV-positive head and neck cancer in the literature and the one most likely to be HPV-specific, as it has been reported to be highly expressed in HPV-positive more often than in HPV-negative HNSCC [301,440,453,454]; on the contrary, the majority of miRNAs are found de-regulated regardless of HPV status. Furthermore, it has been demonstrated that HPV is capable of inducing the secretion of miR-9-rich exosomes in HNSCC cells, while exosomal miR-9 derived from HPV-positive HNSCC cells could significantly enhance cell radiosensitivity, possibly through switching macrophages towards the M1 phenotype [455]. Similarly, exosomal miR-9 derived from HPV-positive HNSCC cell lines has been shown to inhibit TGF-β1 signaling-mediated transformation in fibroblasts, which is also associated with a better patient prognosis [453]. While these findings might offer a possible mechanistic explanation as to why HPV-positive HNSCC are generally more sensitive to therapy than their HPV-negative counterparts, they are also indicative of miR-9′s potential as a rather promising therapeutic target for HNSCC patients.

In regards to predictive miRNA biomarkers for HPV-positive HNSCC, a number of studies have specifically focused on identifying miRNA signatures for patient diagnosis, prognosis, and stratification, for the implementation of more efficient treatment approaches. A 5-miRNA salivary panel, consisting of miR-9, miR-134, miR-196b, miR-210, and miR-455, has demonstrated 65% sensitivity and 95% specificity in discriminating between HPV-positive HNSCC patients and healthy controls, while the combination of miR-191, miR-196b, miR-210, and miR-222 has been found capable of distinguishing between HPV-positive and HPV-negative HNSCC patients [190]. A recently developed distinct 6-miRNA signature (Table 5 and Figure 3b) appears to predict the prognosis of individual HPV-positive patients, based on a risk score formula that takes into account the expression and coefficient of miRNAs in the signature [446]. A comparative study has identified an miRNA signature that is differentially expressed and can discriminate between HPV-positive and HPV-negative OPSCCs, while further in silico functional analysis has suggested that a number of genes associated with the aforementioned miRNAs could be potentially targeted for future therapeutic purposes [456]. Furthermore, a set of 14 extracellular vesicle (EV)-derived miRNAs (Table 5 and Figure 3b) has been associated with the presence of HPV in OPSCC cell lines, suggesting that the particular miRNAs could be indicative of HPV status in OPSCC and might potentially present non-invasive blood or salivary future biomarkers for early patient diagnosis and appropriate stratification [457]. A small EV-derived 11-miR ratio signature (Table 5 and Figure 3b), developed through a novel method named stable variable selection (StaVarSel), has demonstrated 90% sensitivity and 79% specificity, when using a high-accuracy model, and 97% specificity and 54% sensitivity, when using a high-specificity model, in detecting HPV-positive OPSCCs. Since the aforementioned panel constitutes a blood-based (serum) marker, it could potentially be utilized for the diagnosis of HPV-positive OPSCCs, as well as for post-therapy surveillance of patients or the earlier detection of possible disease recurrence [458].

## 5. Clinical Trials Assessing the Utility of miRNAs as Biomarkers for HNC Monitoring

The detection, prediction, and prognosis of HNC is particularly challenging, mainly due to cancer heterogeneity and a significant lack of confirmed molecular biomarkers with high prognostic or diagnostic clinical significance, which could improve patient quality of life and therapeutic approaches. Recent research efforts have focused on the identification of miRNAs that will serve as new biomarkers of prognosis and disease surveillance, and as new therapeutic targets for HNC. 

Although miRNAs are the main subject of several in vitro and in vivo studies, only seven clinical trials are currently ongoing with the aim of identifying diagnostic, prognostic, or therapeutic utility of miRNAs in HNC (Table 6). In these trials, miRNA identification and analysis are either the main study subject or among the secondary study goals. Two of these studies (NCT01927354 and NCT02009852) aim to unravel the significance of miR-29b and other members of the miR-29 family in HNSCC prognosis and pathogenesis. In the third miRNA-based clinical trial (NCT04305366), the investigators plan to explore miRNA signatures of several samples (tissue, blood, and saliva) from both HNSCC patients and healthy individuals and to correlate those with diagnosis and disease progression. Finally, in the last miRNA-targeted clinical study (NCT03953443), the primary objective is the delineation of the association between miRNA expression and miRNA promoter methylation, as well as their prognostic and predictive role in primary HPV-negative HNSCC patients. In all of the other ongoing clinical trials (NCT02869399, NCT04453046, and NCT03843515), miRNA identification and evaluation are mainly intended to the adjuvant evaluation of therapeutic efficacy.

## 6. Conclusions

HNSCC is one of the most frequent malignancies of the upper aerodigestive tract [1]. Despite the new targeted therapies against HNSCC, survival rates have not improved significantly over the last few decades [1]. In this context, it is crucial to develop novel, sensitive, and precise diagnostic and prognostic tools. More than a decade of research has indicated that the aberrant expression of miRNAs deranges the well-regulated RNA complexes in the majority of cancer types. Individual miRNAs or signatures have been considered as suitable biomarkers for clinical applications in cancer prognosis and diagnosis, based on the fact that they exhibit unique and stable expression patterns in cancer tissues [10].

The present review aimed to provide a detailed overview of the current understanding of miRNAs, by summarizing some of the most extensively studied miRNAs that are aberrantly expressed in HNSCC and highlighting their utility as biomarkers. By taking into account that the role of miRNAs is multifunctional, and, as such, that one miRNA can regulate more than one gene targets [15], the scope of this review was to highlight the miRNAs that have so far been associated with evidence of clinical significance. To this purpose, we present the clinical trials that are currently investigating miRNA expression in HNSCC patients and discuss the utility of specific miRNAs as biomarkers for patient stratification in HNSCC. Some of these miRNAs have the potential to serve as reliable biomarkers of diagnosis, prognosis, and therapeutic prediction, in the frame of personalized medicine.

Among the 16 miRNAs that were analyzed in the present review, miR-21 appears to be the most extensively studied and the most consistently upregulated in HNSCC. As it has been associated with poor prognostic outcomes, it could serve as a reliable prognostic biomarker in future clinical applications [55,56]. miR-196a/b and miR-23a, also found upregulated in HNSCC, have been linked to more aggressive cancer types and may therefore confer additional prognostic value [57,261]. Other clinically significant biomarkers seem to include miR-375 and miR-200, as their downregulation is strongly associated with metastasis and worse survival outcomes in HNSCC patients [55,126,132]. miR-29, a consistently downregulated miRNA in HNSCC, is closely correlated with cancer growth and migration [291], and is currently the subject of intense investigation for its potential prognostic significance. Notably, the expression of certain miRNAs (miR-99, -34a, -31, -125a/b, -9, -181a, -155, -146a, -16, and let-7) seems to depend on the HNC subtype and/or the sample type which the miRNAs are extracted from; as such, there is no consistency in the pattern of expression (i.e., exclusively upregulated or downregulated). In such cases, the simultaneous testing of several miRNAs in clinical trials could possibly help to further evaluate and strengthen biomarker significance. MiRNA signatures could provide a more precise approach to the management of HNSCC, leading to the significantly better monitoring of patients.

Additional studies investigating the role of specific miRNAs, individually or as signatures, will undoubtedly shed more light on the utility of miRNAs as biomarkers, leading to improved patient stratification in HNC.

## Figures and Tables

**Table 1:** Summary of the most common upregulated miRNAs reported as potential biomarkers in HNSCC.

miRNA	Tumor Site	Sample Type	Biomarker Role	Reference
**let-7a**	LSCC	Serum	Diagnosis	[298]
**miR-9**	OSCC	Saliva	Prognosis and diagnosis	[299]
	ESCC	Plasma	Prognosis and diagnosis	[300]
	OPSCC	Tumor tissue	Recurrence and detection of HPV+ patients	[301]
**miR-16**	ESCC	Tumor tissue/serum	Early detection and prognosis	[278,302]
**miR-18a**	NPC	Tumor tissue	Prediction metastasis and therapeutic target	[303]
	ESCC	Tumor tissue/plasma	Prognosis, detection, and disease monitoring	[304,305,306]
**miR-19a**	OSCC	Serum/tumor tissue	Diagnosis, prognosis, and therapy	[115,307]
	TSCC	Tumor tissue	Prognosis	[308]
	ESCC	Plasma/tumor tissue	Early detection and prediction of progression-free and overall survival	[304,309]
	LSCC	Tumor tissue	Prognosis and differential diagnosis	[310]
**miR-19b**	ESCC	Plasma/tumor tissue	Prognosis and metastasis prediction	[311,312]
	NPC	Tumor tissue	Prediction reduced patient survival and therapeutic target	[313,314]
**miR-20a**	ESCC	Serum	Diagnosis	[315]
	NPC	Serum/plasma	Detection and prognosis	[316,317]
	OSCC	Tumor tissue	Prognosis	[162]
**miR-21**	OSCC	Tumor tissue/plasma	Prognosis	[36,56,57]
	LSCC	Tumor tissue	Prognosis and prediction of lymph node metastasis	[34]
	TSCC	Tumor tissue	Prediction of chemoresistance	[59]
	OPSCC	Tumor tissue	Prediction and therapy	[318]
**miR-23a**	ESCC	Tumor tissue/plasma	Prognosis and prediction of chemoresistance	[261,264]
	TSCC	Tumor tissue	Prediction of chemoresistance	[265]
**miR-24**	OSCC	Plasma	Prognosis	[319]
**miR-26b**	OPSCC	Tumor tissue	Prediction and therapy	[318]
**miR-29b**	NPC	Serum	Prognosis	[320]
**miR-31**	OSCC	Tumor tissue/plasma/saliva	Prognosis and diagnosis, early detection marker, and disease monitoring	[46,148,154]
	HNSCC	Tumor tissue/serum	Prognosis and diagnosis	[147,321]
	LSCC	Plasma/tumor tissue	Early detection	[155]
**miR-34a**	OPSCC	Tumor tissue	Detection and HPV stratification	[109]
**miR-93**	ESCC	Tumor tissue/serum	Prognosis and prediction of metastasis and radiotherapy	[322,323]
**miR-105**	ESCC/LSCC	Tumor tissue	Prognosis	[324,325]
**miR-106**	ESCC	Tumor tissue	Diagnosis	[11,326]
	OPSCC	Tumor tissue	Prediction and therapy	[318]
**miR-125b**	NPC	Tumor tissue	Prognosis	[327]
**miR-134**	OSCC	Plasma/serum/saliva/tumor tissue	Metastasis detection, prognosis, and diagnosis	[299,328]
	LSCC	Tumor tissue	Prognosis and targeted therapy	[329]
	HNSCC	Tumor tissue/plasma	Prediction of poor survival	[330]
**miR-142**	HNSCC	Plasma/tumor tissue	Prognosis and therapy monitoring, discrimination of HPV-positive patients	[245,331]
	ESCC/OSCC	Tumor tissue	Prognosis	[274,332]
	NPC	Tumor tissue/serum	Potential therapy target, prognosis, and prediction of metastasis	[333,334]
**miR-146a**	OSCC/LSCC	Tumor tissue	Prognosis	[7]
	OSCC	Plasma	Disease detection	[242]
**miR-146a/b**	OPSCC	Tumor tissue	Prediction and therapy	[318]
**miR-155**	HNSCC	Plasma/serum/tumor tissue	Prognosis and therapy	[64,229]
	ESCC, OSCC	Tumor tissue	Diagnosis and prognosis and therapy target	[224,226]
**miR-181a**	OSCC	Plasma	Prognosis of lymph node metastasis	[211]
**miR-182**	OPSCC	Tumor tissue	Prognosis	[335]
**miR-184**	TSCC	Tumor tissue/plasma	Early detection	[336,337]
	OSCC	Tumor tissue/saliva	Diagnosis and prognosis	[338,339]
**miR-186**	HNSCC	Plasma	Prognosis and therapy monitoring	[331]
**miR-187**	OSCC	Tumor tissue/plasma	Diagnosis and metastasis prediction	[340,341]
**miR-195**	HNSCC	Tumor tissue/plasma	Prognosis and therapy monitoring	[331]
	OPSCC	Tumor tissue	Prediction and therapy	[318]
**miR-196a**	OSCC	Tumor tissue/plasma	Diagnosis, early detection, and prediction of recurrence and patients’ survival	[57,171,175]
	HNSCC	Tumor tissue	Prognosis and response to radiotherapy	[173]
	LSCC	Tumor tissue	Diagnosis and therapy	[180]
	TSCC	Tumor tissue	Prediction of lymph node metastasis	[179]
**miR-196b**	LSCC	Tumor tissue	Prognosis and potential therapeutic target	[176,177]
	OSCC	Plasma	Early detection	[171]
**miR-200b**	OSCC	Plasma	Diagnosis	[342]
**miR-203**	HNSCC	Tumor tissue	Diagnosis and metastasis detection	[164,343]
**miR-205**	HNSCC	Tumor tissue/plasma	Prognosis, diagnosis, and metastasis detection	[343,344,345]
	ESCC	Tumor tissue	Diagnosis and therapy	[346,347]
	NPC	Plasma/serum	Disease detection	[316,348]
	OPSCC	Tumor tissue	Prognosis and HPV stratification	[335]
**miR-223**	OSCC	Tumor tissue/serum	Prognosis and therapy target	[349,350]
	NPC	Serum	Prognosis and detection	[351]
	ESCC	Tumor tissue/serum/plasma	Prognosis and diagnosis	[305,352]
	HNSCC	Tumor tissue/plasma	Therapy and disease monitoring	[36]
**miR-372**	OSCC	Tumor tissue	Prognosis and therapy resistance	[162]
	ESCC	Tumor tissue	Prognosis	[353]
	OPSCC	Tumor tissue	Prognosis and metastasis detection	[354]
**miR-373**	OPSCC	Tumor tissue	Metastasis detection	[354]
**miR-374b**	HNSCC	Plasma	Prognosis and disease monitoring	[331]
**miR-423**	OSCC	Plasma/saliva	Early detection and disease monitoring	[355,356]
**miR-424**	OSCC	Serum	Detection	[357]
**miR-483**	OSCC	Serum	Diagnosis and prognosis	[358]
	ESCC	Tumor tissue/serum	Prognosis and therapy target	[359,360,361]
	NPC	Plasma	Prognosis	[362]
**miR-486**	OPSCC	Saliva	Detection and early diagnosis	[363]
**miR-626**	OSCC	Serum/tumor tissue	Prognosis	[364,365]
**miR-1281**	OPSCC	Tumor tissue	Therapy target and metastasis detection	[366]
**miR-3194**	OPSCC	Tumor tissue	HPV patients’ stratification, metastasis detection, and therapy target	[366]
**miR-3651**	OSCC	Whole blood	Prognosis, metastasis monitoring, and detection of the recurrence	[367,368]
**miR-5100**	OSCC	Serum	Prognosis	[364]

miRNA: microRNA; HNSCC: head and neck squamous cell carcinoma; LSCC: laryngeal squamous cell carcinoma; OSCC: oral squamous cell carcinoma; ESCC: esophageal squamous cell carcinoma; OPSCC: oropharyngeal squamous cell carcinoma; TSCC: tongue squamous cell carcinoma; NPC: nasopharyngeal carcinoma; HPV: human papilloma virus.

**Table 2:** Summary of the most common downregulated miRNAs reported as potential biomarkers in HNSCC.

miRNA	Tumor Site	Sample	Biomarker Role	Reference
**let-7a**	HNSCC/OSCC	Saliva/tumor tissue	Early detection and poor prognosis	[117,369]
	ESCC	Serum	Diagnosis	[315]
**let-7b**	ESCC/OSCC	Tumor tissue	Therapy target	[370]
**let-7d**	HNSCC/OSCC	Tumor tissue	Progression	[112,114]
**miR-9**	NPC	Tumor tissue/plasma	Recurrence, metastasis, and disease monitoring	[194,371]
	OSCC	Tumor tissue/serum	Prognosis	[197,372]
	HNSCC	Tumor tissue	Prediction and therapy resistance	[198,373]
**miR-10b**	OPSCC	Saliva	Detection	[363]
**miR-16**	OSCC	Tumor tissue	Tumor suppression and prognosis	[273,275]
	OSCC	Saliva	Early diagnosis	[276]
**miR-24**	NPC	Tumor tissue	Prognosis of recurrence, radioresistance	[374]
**miR-26a**	ESCC	Tumor tissue	Prediction of metastasis	[375]
	TSCC	Tumor tissue	Prognosis	[376]
**miR-26b**	HNSCC	Plasma/Serum	Early detection, prognosis, and therapeutic evaluation	[41,64]
**miR-26a/b**	ESCC/OSCC/TSCC	Tumor tissue	Prevention and therapeutic target	[377,378,379]
**miR-29**	TSCC	Tumor tissue	Progression and therapy target	[295]
	ESCC	Serum	Prediction and radioresistance	[297]
**miR-31**	NPC	Peripheral blood	Tumor suppression, early diagnosis, and therapeutic target	[151]
**miR-34a**	HNSCC	Tumor tissue	Tumor suppression, diagnosis, and therapeutic target	[104,105]
	NPC	Tumor tissue	Prognosis	[110]
**miR-92b**	OSCC	Plasma	Metastasis detection	[42]
**miR-93**	HNSCC	Saliva	Radiotherapy monitoring	[134]
**miR-99**	OSCC	Tumor tissue/serum	Clinical outcome, early detection of disease, and prognosis	[75,87]
	HNSCC	Plasma	Prognosis	[36]
**miR-101**	HNSCC/ESCC	Tumor tissue/serum	Diagnosis and prediction of metastasis	[42,380,381]
	OSCC	Tumor tissue	Prognosis and therapeutic target	[162,382]
	TSCC/LSCC	Tumor tissue	Diagnosis and therapeutic target	[383]
**miR-124**	NPC/ESCC	Tumor tissue/plasma	Prognosis and chemosensitivity	[371,384,385]
**miR-125a**	OSCC	Saliva	Diagnosis and prediction	[133,134]
	HNSCC	Tumor tissue	Prognosis and therapy target	[166]
**miR-125b**	OSCC/HNSCC	Serum/tumor tissue	Prognosis, early diagnosis, and prediction Development and progression	[83,116,162]
	LSCC	Tumor tissue/plasma	Early detection and progression	[159,163]
	ESCC	Tumor tissue	Prognosis	[161]
**miR-130b**	OPSCC	Tumor tissue	Progression and radioresistance	[386]
**miR-139**	OSCC/TSCC	Saliva/tumor tissue/serum	Prognosis, early detection, disease monitoring, therapeutic targeτ	[116,387,388,389]
**miR-146a**	ESCC	Tumor tissue/serum	Tumor suppression and prognosis	[244]
**miR-138**	OSCC/ESCC	Serum/tumor tissue	Early detection, metastasis, and prognosis	[357,390,391]
**miR-150**	ESCC/OSCC	Plasma/tumor tissue	Prediction of overall survival and metastasis and early detection	[328,355,392]
	NPC	Tumor tissue/serum	Diagnosis, prognosis, personalized treatment, and prediction of metastasis	[348,393,394]
**miR-181a**	OSCC	Tumor tissue	Progression	[395]
	LSCC	Tumor tissue	Early diagnosis and treatment target	[210,396]
	ESCC	Plasma/serum	Diagnosis and prognosis	[214,215]
**miR-195**	ESCC	Tumor tissue	Diagnosis, patient classification, and prognosis	[397,398]
**miR-200a**	HNSCC/OSCC	Saliva	Detection and radiotherapy monitoring	[133,134]
**miR-200a/c**	LCSS	Tumor tissue	Prognosis and disease recurrence	[130]
	OSCC	Saliva/oral rinse	Disease detection	[63]
**miR-203**	ESCC	Tumor tissue	Prognosis and detection of metastasis	[390,399]
	OPSCC	Tumor tissue	Prediction and therapy	[318]
	OSCC/TSCC/NPC	Tumor tissue	Prediction and radiotherapy resistance	[400,401]
**miR-204**	HNSCC	Tumor tissue	Prognosis and therapeutic target	[402]
	OSCC/LSCC	Tumor tissue	Prognosis of overall survival and therapeutic target	[175,403,404]
**miR-205**	OSCC/ESCC	Saliva/serum	Diagnosis, prognosis, and disease monitoring	[299,335,405]
**miR-222**	OSCC	Plasma	Early detection	[355]
**miR-223**	LSCC	Serum	Diagnosis	[298]
**miR-375**	HNSCC	Tumor tissue	Prognosis and prediction of metastasis	[68,406]
	LSCC/ESCC	Tumor tissue	Overall survival and prognosis	[34,68]
	OSCC	Plasma	Monitoring recurrence after surgery	[42]
	OPSCC	Tumor tissue	Prognosis	[407]
**miR-486**	OSCC	Plasma/saliva/tumor tissue	Early diagnosis and recurrence	[42,408,409]
	ESCC	Tumor tissue	Prognosis	[410]
**miR-892b**	NPC	Plasma	Monitor recurrence and metastasis	[371]
**miR-3651**	OSCC/ESCC	Tumor tissue	Diagnosis and prognosis	[411,412]

miRNA: microRNA; HNSCC: head and neck squamous cell carcinoma; LSCC: laryngeal squamous cell carcinoma; OSCC: oral squamous cell carcinoma; ESCC: esophageal squamous cell carcinoma; OPSCC: oropharyngeal squamous cell carcinoma; TSCC: tongue squamous cell carcinoma; NPC: nasopharyngeal carcinoma.

**Table 3:** Identified gene hotspots and/or respective pathways and cellular function targeted by miRNAs in HNC.

miRNA	Function	Experimental Set-Up	Target Gene or Signaling Pathway	HNC Subtypes	References	Cellular Functions
**miR-21**	Oncogenic	In vitro	PTEN	OSCC	[45]	Proliferation, invasion, metastasis, apoptosis
			p53		[46]	
			p63		[47]	
			PDCD4		[48,49,50]	
**miR-375**	Onco-suppressor	In vitro	XPR1	ESCC	[61]	Proliferation, migration, invasion
			HNF1β	LSCC	[65]	
			PDK1	NPC	[60]	
			USP1		[66]	
**miR-99**	Onco-suppressor	In vitro	IGF1R mTOR	HNSCC	[77]	Proliferation, migration, invasion
**miR-34a**	Onco-suppressor	In vitro	FLOT-2 MEK/ERK1/2	HNSCC	[105]	Proliferation, migration, invasion, EMT
			SATB2	OSCC	[413]	
**let-7 family**	Onco-suppressor	In vitro	Nanog, K-RAS, CASPASE3, IL-8	HNSCC	[113,123,124]	Proliferation, metastasis, chemosensitivity
**miR-200 family**	Onco-suppressor	In vitro	ZEB1/2, BMI1	OSCC	[135,414]	Proliferation, migration, metastasis, malignant CSC-like properties
**miR-31**	Oncogenic	In silico	FIH	HNSCC	[149]	Angiogenesis
**miR-125a**	Onco-suppressor/Oncogenic	In vitro	ERBB2 and ERBB3/CCR7	HNSCC	[166,415,416]	Proliferation, metastasis, invasion
**miR-125b**	Onco-suppressor	In vitro	HMGA2	ESCC	[161]	Proliferation, migration, invasion
**miR-196a**	Oncogenic	In vitro	ANXA1, MAMDC2, HOXB8, p27 (CDKN1B)	HNSCC/OSCC	[173,174]	Proliferation, migration, invasion, EMT, radioresistance
**miR-196b**	Oncogenic	In vitro	SOCS2, PCDH-17, ANXA1	LSCC/HNSCC	[176,177,178]	proliferation, invasion, apoptosis
**miR-9**	Onco-suppressor	In vitro	CXCR4, Wnt/β-catenin	HNSCC	[373]	Proliferation, colony formation
**miR-181a**	Onco-suppressor	In vitro	MAX/miR-181a/NPM1 pathway, ATF2	LSCC	[210,396]	Proliferation, colony formation, migration, apoptosis
			LNC RNA CCAT1	OSCC/NPC	[395,396]	Proliferation, migration, drug resistance
**miR-155**	Oncogenic	In vitro	CDC73, ARID2	OSCC	[225,229]	Proliferation, invasion, apoptosis
**mir-146a**	Oncogenic	In vitro	IRAK1-NF-κB	ESCC OSCC	[246,417]	Proliferation, apoptosis invasion
	Onco-suppressor	In vitro	Snail Vimentin E-cadherin	ESCC	[244,418]	
**miR-23a**	Onco-suppressor	In vitro	SIX1	OPSCC	[256]	Growth, proliferation, chemoresistance, invasion
	Oncogenic	In vitro	HIF1AN	OSCC	[261]	
	Oncogenic	In vitro	PTEN	ESCC	[262]	
**miR-16**	Onco-suppressor	In vitro/in vivo	TLK1, BCL2L2	OSCC	[273,275]	Proliferation, viability, apoptosis
			BPDE/RAR-β2	ESCC	[278]	
**miR-29**	Onco-suppressor	In vitro/in vivo	LAMC2 ITGA6	HNSCC	[291]	Proliferation, apoptosis, invasion, EMT
			ITGB1	HNSCC	[292]	
			LOXL2	HNSCC	[419]	
			DNMT3B	HNSCC	[420]	
			Sp1/PTEN/p-AKT	TSCC	[295]	
			TIAM1	NPC	[294]	

miRNA: microRNA; OSCC: oral squamous cell carcinoma; ESCC: esophageal squamous cell carcinoma; LSCC: laryngeal squamous cell carcinoma; NPC: nasopharyngeal carcinoma; HNSCC: head and neck squamous cell carcinoma; EMT: epithelial–mesenchymal transition; CSC: cancer stem cell.

**Table 4:** Summary of miRNA signatures and their utility as biomarkers in HNSCC.

miRNA	Tumor Site	Sample Type	Biomarker Role	Reference
miR-383, miR-615, miR-877	HNSCC	Serum	Diagnostic	[277]
miR-9, miR-134, miR-191	HNSCC	Saliva	Diagnostic	[191]
miR-21-5p, miR-28-3p, miR-142-3p, miR-191-5p, miR-186-5p, miR-197-3p, miR-425-5p, miR-590-5p	HNSCC	Plasma	Diagnostic	[331]
miR-204-5p, miR-499a-5p, miR-498-5p, miR-4714-3p, miR-30a-5p, miR-1-5p, miR-548f-3p, miR-518a-3p, miR-155-3p, miR-365a-5p, miR-196b-5p	HNSCC	(Bioinformatic analysis/TCGA)	Prognostic	[24]
miR-99a-5p, miR-758-5p, miR-329-3p, miR-137-3p, miR-1229-3p, miR-3187-3p	HNSCC	Tumor tissue	Prognostic	[427]
let-7c, miR-125b-2, miR-129-1, miR-337, miR-654, miR-99a	HNSCC	Tumor tissue (TCGA)	Prognostic	[428]
miR-21-3p, miR-21-5p, miR-96-5p, miR-429	HNSCC	Tumor tissue	Prognostic	[429]
let-7g-3p, miR-6508-5p, miR-210-5p, miR-4306, miR-7161-3p	HNSCC	Tumor tissue	Prognostic	[430]
miR-142-3p, miR-186-5p, miR-195-5p, miR-374b-5p, miR-574-3p	HNSCC	Plasma	Prognostic	[331]
miR-499a, miR-548k, miR-3619, miR-99a, miR-137, miR-3170, miR-654	HNSCC	Tumor tissue (TCGA)	Prognostic	[431]
miR-16, miR-29b, miR-150, miR-1254, let-7e	HNSCC	Cell lines (TCGA)	Predictive (radiotherapy)	[432]
miR-99a, miR-31, miR-410, miR-424, miR-495	HNSCC	Tumor tissue (TCGA)	Predictive (radiotherapy)	[433]
miR-6510-3p, miR-34c-5p	OSCC	Tumor tissue	Diagnostic	[7]
miR-31-5p, miR-21-5p, miR-125b-5p, miR-99a-5p, miR-100-5p, let-7c-5p, miR-24-3p, miR-30c	OSCC	Saliva (Oral Swirls)	Diagnostic	[434]
miR-196a, miR-196b	OSCC	Plasma	Diagnostic	[171]
miR-218, miR-125b, let-7g	OSCC	Tumor tissue	Prognostic	[435]
miR-127-3p, miR-4736, miR-655-3p	OSCC	Tumor tissue	Prognostic	[436]
miR-21, miR-181b, miR-345	OSCC	Tumor tissue	Susceptibility/risk	[437]
miR-21, miR-375	TSCC	Oral (Brush) Cytology, tumor tissue	Diagnostic (screening)	[49]
miR-142-3p, miR-31, miR-146a, miR-26b, miR-24, miR-193b	OPSCC	Tumor tissue	Prognostic	[438]
miR-27a-3p, miR-455-5p, miR-203a-3p, miR-584-5p, miR-24-3p, miR-548k, miR-126-3p, miR-126-5p, miR-365a-5p, miR-98-5p, miR-151b, miR-361-3p, miR-374c-5p, miR-150-5p, miR-374b-5p, miR-107, miR-125b-5p, miR-1287-5p, miR-146a-5p, miR-106a-5p, miR-15b-5p, miR-20b-5p, miR-532-3p, miR-361-5p, miR-363-3p, miR-625-3p	OPSCC	Tumor tissue	Prognostic	[439]
miR-107, miR-151, miR-492	OPSCC	Tumor tissue	Prognostic	[440]
miR-20b, miR-107, miR-151, miR-182, miR-361	OPSCC	Tumor tissue	Prognostic	[440]
miR-151, miR-152, miR-324-5p, miR-361, miR-492	OPSCC	Tumor tissue	Prognostic	[440]
miR-449a-5p, miR-6510-3p, miR-133a-5p	LSCC	Tumor tissue	Diagnostic	[7]
miR-31-3p, miR-196a-5p	LSCC	Plasma	Diagnostic	[155]
miR-200a-3p, miR-30b-5p, miR-4451	HSCC	Tumor tissue	Prognostic	[441]
miR-29c, miR-30e, miR-93	NPC	Tumor tissue (GEO)	Prognostic	[442]
ebv-miR-BART19-3p, miR-135b, miR-141	NPC	Tumor tissue (GEO)/cell line	Prognostic	[443]
miR-142-3p, miR-29c, miR-30e	NPC	(Bioinformatic analysis/GEO)	Prognostic	[334]

miRNA: microRNA; HNSCC: head and neck squamous cell carcinoma; LSCC: laryngeal squamous cell carcinoma; OSCC: oral squamous cell carcinoma; ESCC: esophageal squamous cell carcinoma; OPSCC: oropharyngeal squamous cell carcinoma; TSCC: tongue squamous cell carcinoma; NPC: nasopharyngeal carcinoma; TCGA: The Cancer Genome Atlas; GEO: gene expression omnibus.

**Table 5:** Summary of the most common miRNA signatures associated with HPV infection in HNSCC patients.

miRNA	Tumor Site	Sample Type	Biomarker Role	Reference
miR-9, miR-134, miR-196b, miR-210, miR-455	HNSCC	Saliva	Early-stage detection and HPV+ patients’ stratification	[190]
miR-191, miR-196b, miR-210, miR-222	HNSCC	Saliva	HPV+ patients’ stratification	[190]
miR-378a-3p, miR-16-1-3p, miR-493-3p, miR-380-5p, miR-376c-3p, miR-338-5p	HNSCC	Tumor tissue (TCGA/GDAC)	The prognosis and detection of HPV+ patients	[446]
miR-324-5p, miR-4764-3p, miR-107, miR-1234, miR-3144-3p, miR-3176, miR-3177-3p, miR-4267, miR-4418, miR-615-3p, miR-668, miR-99b-3p, miR-675-3p, miR-584-5p, miR-212-3p, miR-18b-5p, miR-18a5p, miR-138-1-3p, miR-135b-5p, miR-1246, miR-4764-3p, miR-857, miR-7-2-3p	OPSCC	Tumor tissue	The detection of HPV+ patients	[456]
miR-363-3p, miR-551b-3p, miR-20b-5p, miR-20b-3p, miR-143-3p, miR-106a-5p, miR-9-5p (miR-9-1), miR-9-5p (miR-9-2), miR-9-5p (miR-9-3), miR-99a-3p, miR-99a-5p, miR-9-3p (miR-9-1), miR-193a-5p, let-7b-3p	OPSCC	Cell line	Early detection and patient stratification	[457]
miR-206/miR-494-3p, U6 snRNA/miR-150-5p, miR-532-3p/miR-574-3p, miR-125a-5p/miR-193b-3p, miR-1274b/miR-27a-3p, miR-494-3p/miR-150-5p, miR-193a-5p/U6 snRNA, miR-27a-3p/miR-93-5p, ath-miR-159a/miR-152-3p, ath-miR-159a/miR-494-3p, miR-375-3p/miR-483-5p	OPSCC	Serum (EVs)	The detection of HPV+ patients	[458]

miRNA: microRNA; HNSCC: head and neck squamous cell carcinoma; OPSCC: oropharyngeal squamous cell carcinoma; TCGA: The Cancer Genome Atlas; GDAC: Genome Data Analysis Centers; HPV: human papilloma virus; EVs: extracellular vesicles; snRNA: small nuclear RNA.

**Table 6:** Current clinical trials assessing miRNAs’ potential utility as biomarkers.

Identifier	Official Title	Clinical Phase	Target miRNAs	Sample Type	Purpose	Ref.
NCT04305366	MicroRNA Markers in Head and Neck Cancers	N/A	N/D	Fine needle aspiration biopsy, serum, saliva	To investigate the miRNA signature of samples and to develop biomarkers for surveillance of HNSCC patients.	[459]
NCT02869399	A Randomized Phase II Study for Tertiary Prevention of Squamocellular Cancer of Head and Neck (SCCHN) With a Dietary Intervention	II	N/D	Saliva, plasma	To investigate the role of diet as a risk factor for HNSCC recurrence and secondary tumor development, to identify saliva and plasma miRNAs, and to evaluate their change in inflammatory cytokine profile during the course of dietary intervention.	[460]
NCT03953443	INST 1008: Expression and Epigenetic Silencing of MicroRNA for Predicting the Therapeutic Response and Prognosis of HPV- negative Head and Neck Squamous Cell Carcinoma (HNSCC)	N/A	N/D	Tumor tissue, normal tissue	To assess the association between miR expression and miR promoter methylation and the response to therapy and prognosis in primary HPV-negative HNSCC patients.	[461]
NCT04453046	Depleting Exosomes to Improve Response to Immune Therapy in Head and Neck Squamous Cell Cancer: An Early Feasibility Phase I Clinical Trial	I	N/D	Blood	To determine whether the use of Hemopurifier before treatment with pembrolizumab is low-risk and well-tolerated by the patients and whether it leads to reduced levels of exosomes in the blood and to identify immunoinhibitory proteins and miRNA profiles for the evaluation of effectiveness of combination treatment in decreasing immune suppression in patients with recurrent/metastatic HNSCC.	[462]
NCT03843515	Safety and Tolerability of Neoadjuvant Nivolumab for Locally Advanced Resectable Oral Cancer Combined With [18F] BMS-986192/[18F]-FDG PET Imaging and Immunomonitoring for Response Prediction	I	N/D	Tumor tissue, plasma	To further evaluate tumor PD-L1 expression as a predictive biomarker and to investigate the immunophenotype of the patient and tumor, as well as the presence of neoantigens and other potential biomarkers such as plasma vesicle miRNAs.	[463]
NCT01927354	Observational Study on the Investigation of the Molecular Mechanism and Clinical Significance of the Interplay Between Twist1 and Other EMT Regulators Through microRNA-29 Family	N/A	miR-29 family	Tumor tissue	To delineate the regulatory mechanism of the Twist1-miR-29s-SIN3A axis, to investigate the molecular interplay between Twist1 and Snail through Twist1-miR-29s-SIN3A signal pathway, and to elucidate the molecular basis and pathophysiologic significance of Twist1-Snail interaction under hypoxic environment.	[464]
NCT02009852	The Role of microRNA-29b in the Oral Squamous Cell Carcinoma	N/A	miR-29b	Tumor tissue, serum, saliva	To identify a prognostic significance for miR-29b in oral cancer.	[465]

N/A: not available; N/D: no data; HNSCC: head and neck squamous cell carcinoma; miR: microRNA; HPV: human papilloma virus; PET: positron emission tomography; EMT: epithelial–mesenchymal transition.

## Data Availability

Not applicable.
